# First confirmed complete incubation of a flapper skate (*Dipturus intermedius*) egg in captivity

**DOI:** 10.1111/jfb.14816

**Published:** 2021-06-19

**Authors:** Steven Benjamins, Georgina Cole, Adam Naylor, James A. Thorburn, Jane Dodd

**Affiliations:** ^1^ Scottish Association for Marine Science, University of the Highlands and Islands Oban UK; ^2^ Royal Zoological Society Scotland Edinburgh UK; ^3^ Scottish Oceans Institute, University of St Andrews St Andrews UK; ^4^ NatureScot Oban UK

**Keywords:** development, *Dipturus intermedius*, egg, embryo, flapper skate, temperature, time

## Abstract

An egg of the critically endangered flapper skate *Dipturus intermedius* was successfully incubated to hatching in captivity in what is believed to be a first for the species. Water conditions (temperature, salinity, flow rate) were recorded, with mean water temperatures ranging from a monthly mean of 8.3 ± 1.2 to 13.2 ± 0.3°C and salinity from a monthly mean of 30.5 ± 1.2 to 36.6 ± 2.3 ppt. Hatching occurred after 534 days, suggesting that flapper skate eggs take *c*. 5700 growing degree‐days to incubate to hatching. The egg's prolonged embryonic development raises concerns about flapper skate eggs' vulnerability to anthropogenic disturbance.

## 1 INTRODUCTION

**FIGURE 1:**
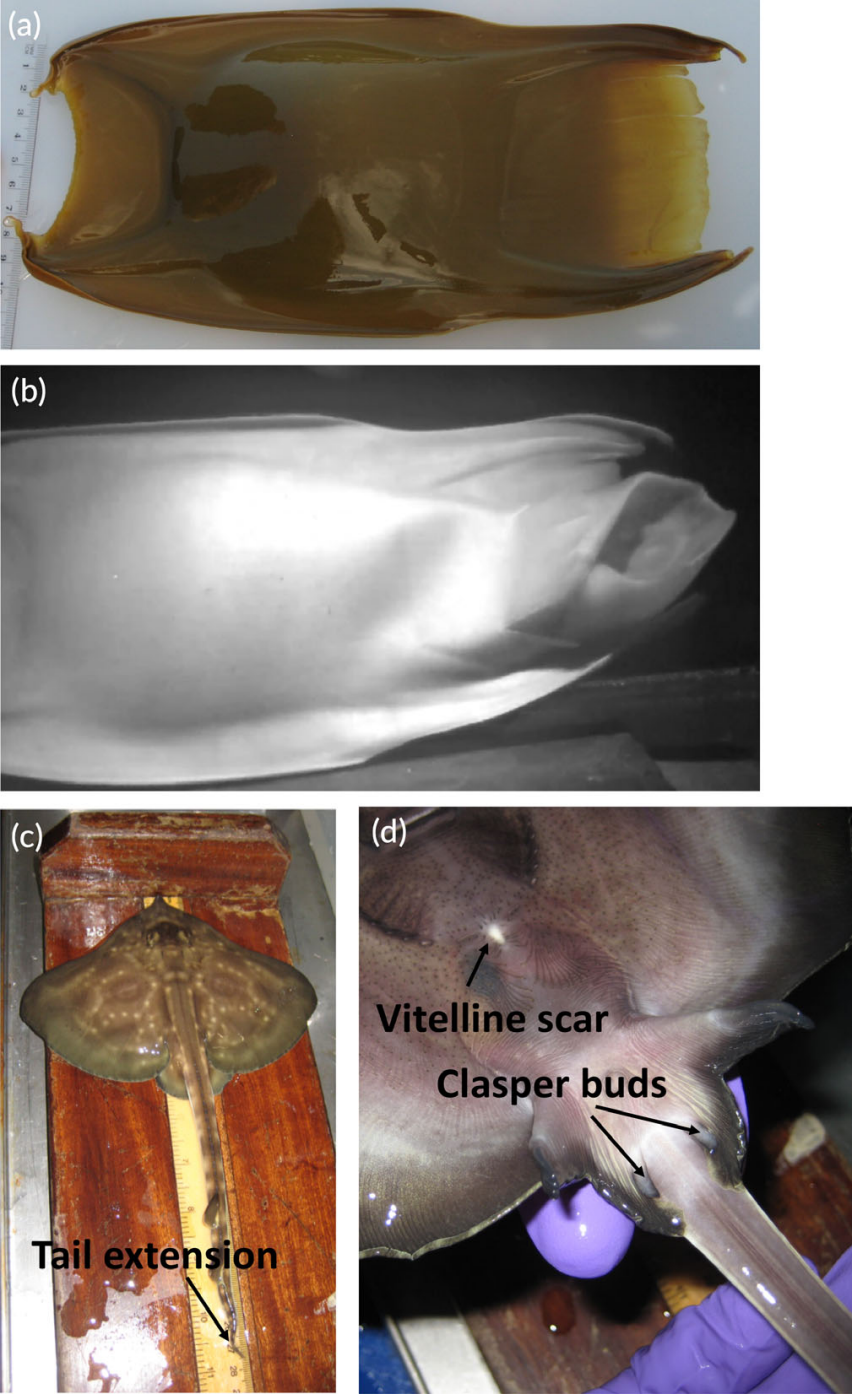
Images of the skate egg and the hatchling described in this paper. (a) The skate egg. (b) The moment the skate hatchling emerged from the egg (facing right), wings still folded above the body (a video of the hatching is available at https://www.youtube.com/watch?v=j8Ewgg_INRs). (c) Dorsal view of the hatchling being measured. Note the whip‐like extension to the tail (black arrow). (d) A view of the hatchling's underside, showing the vitelline scar and the clasper buds alongside the pelvic fins, indicating it was a male (black arrows)

The flapper skate (*Dipturus intermedius*) is a large demersal batoid found in the northeast Atlantic (Dulvy *et al*., 2006). Formerly part of what is known as the “common skate complex,” the flapper skate is now known to be genetically distinct from the common blue skate (*Dipturus batis*; Iglésias *et al*., 2010; Last *et al*., 2016). This species complex is listed as critically endangered on the IUCN Red List (Dulvy *et al*., 2006) due to a reduction in the species' range following a long period of overexploitation (Brander, 1981). The total length of the flapper skate is reported as “up to 230 cm, possibly 285 cm for females,” which grow larger than males (Ebert & Stehmann, 2013). Their egg cases also rank among the largest skate egg cases at 100–144 mm width and 130–235 mm length, excluding the short horns (Figure 1a; Gordon *et al*., 2016).

Understanding the developmental biology of long‐lived, slowly reproducing species such as skate is important to accurately understand risks and implement appropriate conservation measures across the species' life cycle. What little is known about flapper skate embryonic development is confused by the split of the common skate species complex in 2010 (Iglésias *et al*., 2010). Literature pre‐dating the species complex split describes common skate embryos as taking from 2 to 5 months (Little, 1995) to “possibly years at high latitudes” (Ebert & Stehmann, 2013) to incubate to hatching; skate hatchlings have been reported to measure 21–29 cm long (Ebert & Stehmann, 2013). Detailed studies have been published on the incubation period and size at hatching of other *Dipturus* species (Concha *et al*., 2018; Parent *et al*., 2018), including *Dipturus batis* (9–10 months; Beard, 1890, Clark, 1922), but to date no comparable data have been available on flapper skate, representing a significant data gap.

The influence of temperature on growth rates of individuals and incubation periods of eggs underpins the concept of growing degree‐days (GDDs, Neuheimer & Taggart, 2007). GDDs describe egg incubation periods based on the average temperature during incubation, multiplied by the number of days to hatching, and are used to compare incubation periods both within single species at different temperatures (Embody, 2011) and between species (Chezik *et al*., 2014). Although hatching times among eggs in the same fertilisation batch may vary across a wide range of GDDs, the average number of GDDs is relatively constant for each particular species (Neuheimer & Taggart, 2007). This study aimed at reporting an incubation period for an opportunistically collected flapper skate egg expressed in GDDs.

## 2 MATERIALS AND METHODS

On 4 April 2019, a female flapper skate was captured by a recreational sea angling charter in the northern Sound of Jura, western Scotland (UK). While on deck, the female released a fully developed egg, which was retained in sea water as per the lead author's request and quickly transported 44 km from Ardfern marina to the aquarium facility at the nearby Scottish Association for Marine Science (SAMS) inside a cool box filled with sea water. Due to the opportunistic nature of this sample, the egg was first stored outside in a shaded aquarium tank for 3.5 days before being transferred inside on 8 April 2019 into an insulated and darkened aquarium that became known as the “development tank.” A constant amount of sea water was supplied to the tank, which was pumped from the outside environment *via* a sub‐sand intake (placed 70 cm below the seabed surface at *c*. 0.3 m below Lowest Astronomical Tide, *i.e*., within *c*. 5 m of the sea surface at all times) and allowed to settle. Before entering the development tank, the water passed through a chiller, manually set at 1–2°C below the temperature of the water entering the facility, although due to a fault noted below, this was not maintained throughout the study. An airstone was placed in the tank to aid oxygenation.

In August 2020 the egg was transferred to a new tank (maintaining the same water quality standards) which became known as the “camera tank” to allow for video recording. This tank was kept dark by attaching black corrugated plastic to the outside. The egg was propped up against a rock to allow the camera to be focused on the anterior end of the egg to capture the moment of hatching and was recorded continuously using a macro camera (Axis Communications, model P1357, Stockholm, Sweden) with infrared illuminator (model: IR 70; 940 nm) through a hole in the plastic. Water temperature (using a hand‐held mercury thermometer), salinity (using an OPTI Aquatic digital hand‐held refractometer followed by a Brix portable refractometer when the OPTI unit malfunctioned on 22 June 2020) and water inflow rate inside both the development tank and the camera tank were recorded several times each week for the duration of the embryonic development. Average monthly temperature was calculated to account for seasonal variation in temperature and multiplied by the number of days in the month to calculate GDDs each month. These were added together to calculate the total number of GDDs.

Once weekly, the egg was transferred to a small, clear, plastic container, which was then placed on a light box for up to 5 min to illuminate the egg from below to evaluate embryonic development; care was taken to ensure it remained submerged at all times. The egg case was imaged using a downward‐facing mounted camera (Panasonic DMC‐FT30, 180 dpi, Tokyo, Japan) following the method used by Musa *et al*. (2018), before being returned to its tank. Ultrasound scanning of the egg was undertaken opportunistically on four occasions, using three different units: A Sonoscape E1V with a micro‐convex 4–13 MHz probe (18 August 2019), an Easi‐Scan veterinary scanner with a micro‐convex 4.5–8.5 MHz probe (19 November 2019 and 17 April 2020) and a Clarius C3 HD Vet ultrasound with a convex 2–6 MHz probe (9 September 2020). The egg remained submerged during sonographic imaging.

## 3 RESULTS

**FIGURE 2:**
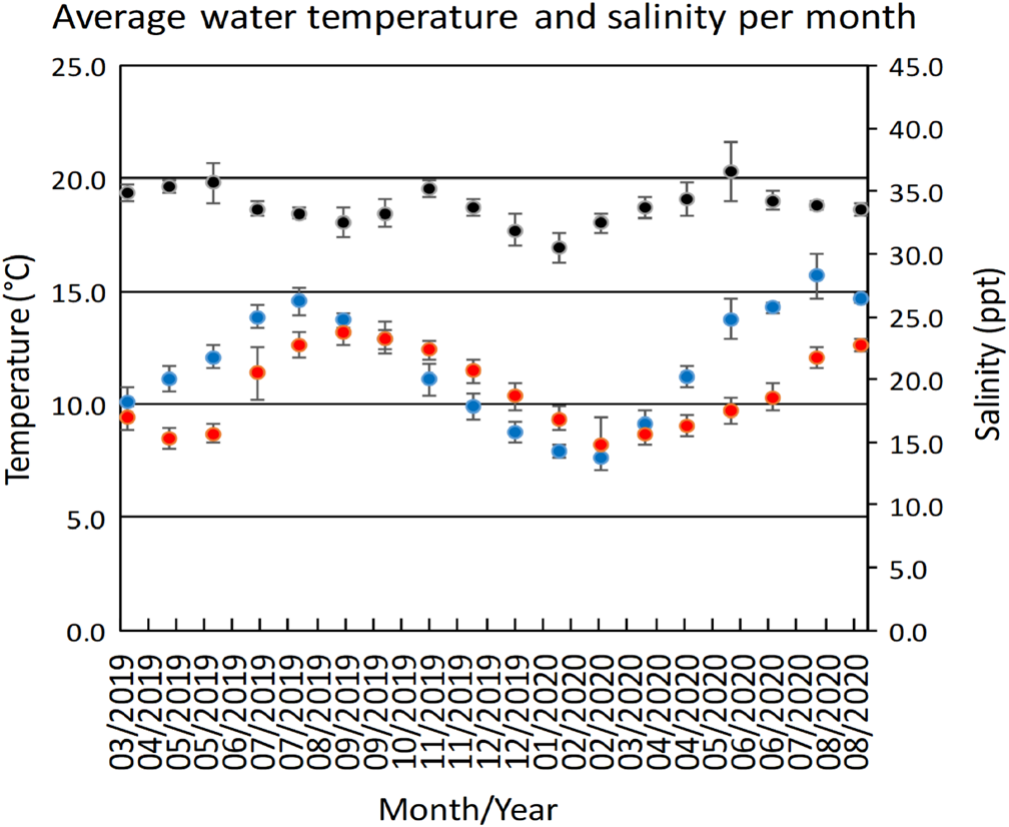
Average monthly temperature and salinity profile experienced by the skate egg. From 24 August 2020, the egg was permanently transferred from the “development tank” to the “camera tank” for constant observation. 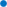, Average incoming water temperature (°C); 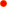, Average temperature in tank (°C); 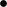, Average salinity in tank (ppt)

During the course of development, monthly mean salinity varied between 30.5 ppt (s.d. ± 1.2 ppt) and 36.6 ppt (s.d. ± 2.3 ppt), with a long‐term mean of 33.6 ppt (s.d. ± 1.7 ppt), responding to local rain runoff. Monthly mean water inflow rates varied between 11.8 l h^–1^ (s.d. ± 5.8 l h^–1^) and 44.2 l h^–1^ (s.d. ± 5.4 l h^–1^) (mean 21.8 l h^–1^; s.d. ± 7.1 l h^–1^) due to periodic debris build‐up inside pipes and differences in supply rates between the development tank and the camera tank. Monthly mean water temperatures varied between 8.2°C (s.d. ± 1.2°C) and 13.2°C (s.d. ± 0.5°C); the overall mean water temperature in the tank was 10.9°C (s.d. ± 1.7°C) (Figure 2). Due to a fault in the chiller's temperature controls, the water temperature in the development tank during September 2019–March 2020 was not 2°C cooler than the temperature of the water entering the aquarium facility as planned but rather 2°C warmer.

**FIGURE 3:**
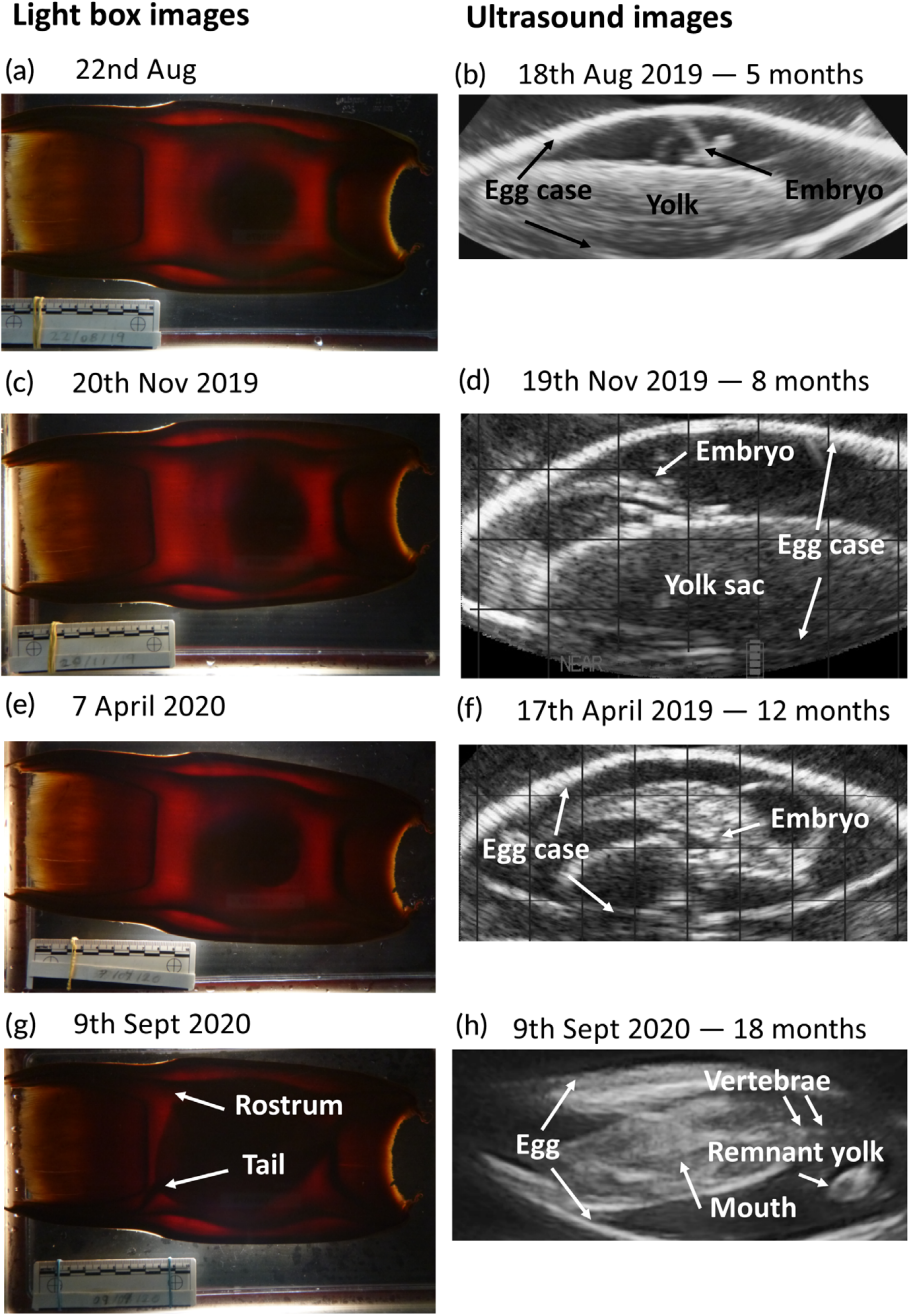
Light box and ultrasound images at 5, 8, 12 and 18 month development. Gridlines (where included) represent 10 mm × 10 mm. (a) Image recorded when egg was photographed on 22nd Aug 2019. (b) Ultrasound image of egg recorded on 18th Aug 2019, 5 months after laying. (c) Image recorded when egg was photographed on 20th Nov 2019. (d) Ultrasound image of egg recorded on 19th Nov 2019, 8 months after laying. (e) Image recorded when egg was photographed on 7th April 2020. (f) Ultrasound image of egg recorded on 17th April 2020, 12 months after laying. (g) Image recorded when egg was photographed on 9th Sept 2020. (h) Ultrasound image of egg recorded on 9th Sept 2020, 18 months after laying

The first evidence of a developing embryo, distinct from the yolk sac, was recorded *via* ultrasound on 18 August 2019 (Figure 3). As time passed, the embryo assumed a recognisable batoid shape that started to obscure the shrinking yolk sac (from at least January 2020 onwards). The last photograph before hatching was taken on 16 September 2020. This shows the embryo filling the egg capsule, with the tail visible in the left corner of the anterior end of the egg case while the rostrum is visible in the posterior left corner (Figure 3g). This is similar to observations of pre‐hatching embryos in other skate species as described by Hoff (2009) and others. During the ultrasound on 19 November 2019, the anterior filament of the egg was recorded as moving independently of water flow. This suggested that the horns of the egg case had opened, allowing the embryo to create a flow of water through the egg; ultrasound imaging confirmed this. The stages described in Musa *et al*. (2018), stage 1 (ellipsoid yolk, no embryo, Figure 3a), stage 3 (development of a long tail and >60% of the embryo body not connected to the surface of the yolk, Figure 3b) and stage 7 (final stage, Figure 3h), were identifiable from the ultrasound images and the timing of events (stage 1 being newly laid and stage 7 being fully developed).

The hatchling emerged from the egg at 22:47 GMT on 19 September 2020, or 534 days (*c*. 18 months) after being laid, equivalent to 5692 GDDs. The hatchling quickly forced its way through the anterior apron of the egg (within 15 s) and unfurled its wings that were folded dorsally (Figure 1b) in a similar way to other skate species (*e.g*., Luer & Gilbert, 1985). The male hatchling measured 279 mm total length and 184 mm disc width and weighed 89 g (Figure 1c). It displayed a bilaterally symmetrical pattern of lighter‐coloured spots arranged in rough polygons on the dark‐brown dorsal surface, similar to adult flapper skate; the spots appeared less distinct than those observed in adult skate, suggesting ontogenetic changes to dorsal patterning (Benjamins *et al*., 2018, Figure 1c). The ventral surface was almost black, with white patches on the middle of the wings and on the pelvic fins (Figure 1d). The hatchling also displayed the temporary whip‐like extension to the tail (Figure 1c).

## 4 DISCUSSION

Recreational angling presents a unique opportunity to obtain flapper skate eggs close to their natural deposition state. Nonetheless, spontaneous release is rare, which limited the number of eggs collected in this study to one. Although the act of handling the female may have caused the egg to be deposited earlier than would have been expected, the egg appeared to be fully formed and hardened when it was released on to the deck of the vessel, suggesting it was near term. This study was undertaken opportunistically, and as such, the authors acknowledge lessons learned for future work.

Temperature has been shown to impact the intra‐species incubation times of elasmobranch eggs markedly. For example, Hume (2019) reported 12%–23% increases in development rates in water temperatures 2–4°C warmer for *Raja microocellata*. Therefore, to accurately determine embryonic development time for flapper skate, the temperature in the development tank should have reflected that experienced in the wild. Due to a technical fault, temperature was not maintained at seasonal norm throughout the development of the egg; therefore, the natural development time of flapper skate embryos is likely to vary around the 534 days reported here. The incubation time of 534 days is significantly longer than that reported for smaller‐bodied *Dipturus* species; *c*. 252 days for *Dipturus chilensis* (Concha 2019) and 9–10 months for *D. batis* (blue skate; Beard, 1890) but is comparable to development time reported for larger species (*e.g*., 342–494 days for *Dipturus laevis*; Parent *et al*., 2018), lending confidence to the development time reported in this study.

Salinity also varied in the development tank and the camera tank, resulting in an interesting incidental observation that flapper skate embryos appear tolerant to such fluctuations in salinity. In addition, the repeated movement of the egg for weekly monitoring may have influenced the development time of the embryo; all efforts were, however, made to minimise any impact of this disturbance. Therefore, several caveats should be noted when considering the development time reported here, not least that only a single egg was available for study.

It was not possible to identify any of the development stages described by Musa *et al*. (2018) based solely on the light box images. This was due to the flapper skate egg case's opaque nature, the batoid shape of the embryo obscuring the gills and the large size of the yolk obscuring the embryo. Therefore, the backlighting approach cannot presently be recommended to determine the exact developmental stage of flapper skate eggs in the field, either underwater or on the surface. Ultrasound imaging proved to be a useful non‐invasive tool to determine the viability of the embryo during early development stages.

The significant development time of flapper skate eggs reported here increases their chances of interactions with damaging anthropogenic activities. This highlights the urgency to identify and protect preferred habitats for flapper skate egg laying to support recovery of the species.

## CONFLICTS OF INTEREST

The authors declare that they are not aware of any competing interests.
